# Diagnostic Laboratory Testing and Clinical Preparedness for Dengue Outbreaks during the COVID-19 Pandemic

**DOI:** 10.4269/ajtmh.20-0884

**Published:** 2020-07-28

**Authors:** Stephen H. Waterman, Gabriela Paz-Bailey, Jose L. San Martin, Gamaliel Gutierrez, Luis G. Castellanos, Jairo A. Mendez-Rico

**Affiliations:** Dengue Branch; Division of Vector Borne Diseases; Centers for Disease Control and Prevention; San Juan, Puerto Rico; E-mails: shw2@cdc.gov, gmb5@cdc.gov; Neglected, Tropical and Vector Borne Diseases Unit; Department of Communicable Diseases and Environmental Health Determinants; Pan American Health Organization; Washington, District of Columbia; E-mails: sanmartij@paho.org, gutierrezg@paho.org, castellanosl@paho.org; Infectious Hazard Management; Health Emergencies Department; Pan American Health Organization; Washington, District of Columbia; E-mail: ricoj@paho.org

Dear Sir,

Wilder-Smith and coauthors made key observations regarding the importance of maintaining mosquito control and clinical preparedness for dengue during the COVID-19 pandemic crisis, especially given the resurgence of dengue in Latin America in 2019 and 2020.^1–3^ Clearly, simultaneous outbreaks of COVID-19 and dengue pose high potential for overwhelming healthcare systems; and because the two diseases can have similar nonspecific clinical presentation in early stages, alerting clinicians and putting in place carefully drawn clinical algorithms for triage are critical to reducing mortality. We would like however to qualify and elaborate on Wilder-Smith et al.’s comment regarding virologic and diagnostic testing in this context.

Because diagnostic laboratory testing for dengue is mostly conducted for surveillance purposes in the Americas and is typically not performed in clinical settings, dengue diagnosis is primarily based on clinical presentation and disease progression.^4^ Whereas molecular testing for COVID-19 can be performed in most Latin American countries, laboratory testing for COVID-19 may not be frequently available because of limited resources, results may be delayed, and COVID-19 rapid tests may lack sensitivity and specificity.^5^ Although we are skeptical that dengue antibodies cross-react with COVID-19 virus, rapid diagnostics tests for dengue, though potentially useful, may lack sensitivity and specificity.^6,7^

Given limitations in diagnostic testing for both dengue and COVID-19, suspect febrile illnesses in dengue-endemic areas require patient/family guidance to monitor warning signs of severe illness and to seek evaluation and care if signs of symptoms of either disease occur. Patient education and clinical triage protocols should note that the critical period for the most frequent severe complication of dengue, shock due to capillary leakage, can occur somewhat earlier in the course of illness (3–7 days after fever onset, around the time of defervescence) than does the respiratory decompensation seen in COVID-19 patients (5–8 days). Thus, clinicians in dengue-endemic areas during the COVID-19 pandemic should remain vigilant for warning signs of potentially severe dengue illness including abdominal pain, persistent vomiting, bleeding, and lethargy or restlessness, and communicate counseling messages regarding these warning signs to patients with suspected early-stage COVID-19, particularly during dengue outbreaks.^4^ Furthermore, clinicians should recognize that some of the dengue clinical warning signs may also be seen in COVID-19 cases.^8^
